# P38γ Promotes Tumorigenesis Through Activating Immune Evasion

**DOI:** 10.3390/cells15131226

**Published:** 2026-07-07

**Authors:** Naveenkumar Chandrashekar, Xiao-Mei Qi, Guan Chen

**Affiliations:** 1 Department of Pharmacology and Toxicology, Medical College of Wisconsin, 8701 Watertown Plank Road, Milwaukee, WI 53226, USA; nchandrashek@mcw.edu (N.C.); xiaoqi@mcw.edu (X.-M.Q.); 2 Research Service, Clement J. Zablocki Veterans Affairs Medical Center, Milwaukee, WI 53295, USA

**Keywords:** p38γMAPK, breast cancer, pancreatic cancer, colon cancer, immune survival, PD-L1

## Abstract

Stress MAPKp38γ (*MAPK12*) has established roles in promoting tumorigenesis; however, the mechanisms involved remain largely unclear. This paper will review recently published and unpublished studies of p38γ in programming immune evasion in breast cancer, pancreatic cancer, and colon cancer to promote tumorigenesis. First, we show that p38γ is an oncogene that transforms breast epithelial cells into triple-negative breast cancer (TNBC), is required for breast tumorigenesis in mice, and activates tumor-suppressive environments via a positive feedback signaling loop. Moreover, we show that epithelial p38γ is required for KRAS-oncogene-induced pancreatic cancer in two genetic murine models (KPC and KTC) by activating glycolytic pathways to provide metabolic support for cancer cells and by increasing chemokine CXCL5-dependent fibrosis and immune cell infiltrations. Lastly, we will delineate how p38γ is activated by the main risk factors for colon cancer and serves as a key integrator of oncogenic and inflammatory signaling to promote tumorigenesis by increasing Wnt proliferative signaling and programming immune evasion. These results indicate that p38γ MAPK can integrate common risk factors for colon cancer and amplify oncogenic signaling by phosphorylating its substrate, β-catenin, increasing transcription of Wnt and the chemokine CXCL13, and promoting PD-L1 expression. In each of these tumor models, we will present evidence supporting our hypothesis, followed by additional experiments for verification. Our studies suggest that targeting p38γ may be an innovative approach in cancer therapeutic intervention.

## 1. Breast Cancer

Role of p38γ (gene name: *MAPK12*) in breast cancer: p38γ regulates cytokine/chemokine expression, is involved in stress response and inflammation-associated cancer, and its expression level is higher in triple negative breast cancer (TNBC) that lacks expression of estrogen receptor (ER), progesterone receptor (PR), and human epidermal growth factor receptor 2 (HER2) than non-TNBC [1]. In ER+ breast cancer cells, p38γ binds nuclear ER and antagonizes ER RNA and protein expression [2] and phosphorylates ER at S118, thereby switching ER signaling from the classical to the nonclassical pathway, thus increasing breast cancer hormone sensitivity [3]. p38γ-forced expression also phosphorylates DNA topoisomerase-IIa at S1542, leading to its stabilization and increased growth inhibition of breast cancer sensitivity by Topo II inhibitors [4]. Therefore, p38γ can activate and modify the therapeutic target activity in breast cancer cells through phosphorylation.

**Figure 1:**
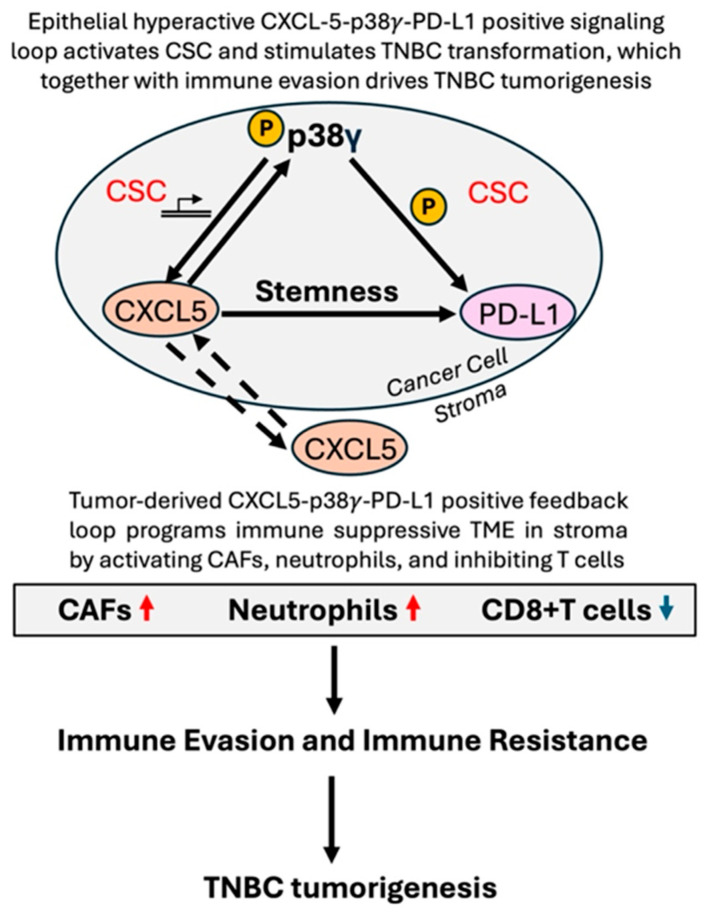
The p38γ/CXCL5/PD-L1 signaling loop in TNBC tumorigenesis. p38γ promotes TNBC progression through a positive feedback loop involving CXCL5 and PD-L1, enhancing CSC expansion (cancer stemness), neutrophil recruitment, and immune evasion while reducing CD8+ T-cell infiltration. This signaling axis cooperatively drives tumor growth and therapy resistance, suggesting that combined inhibition of p38γ, CXCL5, and PD-L1 could represent a promising therapeutic strategy for TNBC.

p38γ significantly promotes cancer-like stem cell (CSC) transformation in TNBC through its nuclear activity, potentially shaping an immunosuppressive tumor microenvironment (TME). It binds strongly to c-Jun at the AP-1 site of the MMP9 [5] and Nanog promoters [6], leading to increased breast cancer invasion and metastasis, CSC stimulation [7], and reduced hormone sensitivity in breast epithelial cells [3]. Overexpression of p38γ in human breast epithelial MCF10A cells can induce TNBC transformation by activating CSCs, while silencing p38γ in TNBC cells reduces stemness, tumor growth, and metastasis, suggesting that p38γ drives TNBC development by promoting CSC expansion and establishing an immunosuppressive TME. Recent bioinformatic and genetic studies support this, showing that p38γ overexpression is involved in pan-cancer pathways that increase cell proliferation and regulate immune suppression }via activation of the chemokine CXCL5 and the checkpoint protein PD-L1. Although PD-L1 enhances CSC activity and TNBC progression [4,8] and CXCL5 facilitates metastasis [8] and immunosuppression [9], our findings reveal, for the first time, that p38γ interacts with PD-L1 and CXCL5 to promote TNBC by stimulating CSCs and programming an immunosuppressive environment. This discovery suggests a novel mechanism of TNBC tumorigenesis, which could offer new therapeutic targets (Figure 1).

TNBC is the most aggressive form of breast cancer, lacking expression of the ER, PR, and HER2, making it difficult to treat. About 75% of TNBC cases are classified as basal-like breast cancer. It has the poorest prognosis, with a metastatic 5-year survival rate of only about 12%, mainly due to the absence of targeted therapies. Although immunotherapy with checkpoint inhibitors has emerged as a promising breakthrough, responses are often limited by innate resistance [10]. TNBC is characterized by an abundance of CSCs [11] surrounded by dense stroma, including cancer-associated fibroblasts (CAFs) [12] and immune cells, which together create an immunosuppressive TME. This promotes tumor progression and resistance to treatment. Developing therapies that target both CSCs and the immunosuppressive TME could improve outcomes for TNBC patients and is an urgent clinical need [13].

Chemokines are 8–12 kDa proteins crucial to tumor-stroma communication [14]. They transmit signals from tumor to stromal cells, shaping the TME [15]. Their functions can be both anti- and pro-tumorigenic, influenced by tumor stage, the immune cells involved, and receptor expression and distribution [16]. Specifical C-X-C motif chemokine ligand 5 (CXCL5) correlates with breast cancer severity and, along with its receptor CXCR2, promotes tumor-promoting activities and metastasis [8]. Although CXCL5 facilitates tumor–host interactions [16], recruits neutrophils, promotes fibrosis, and activates CSCs—aiding immune evasion and pre-metastatic niche formation and metastasis [8], the signaling pathways that drive its gene expression remain largely unclear.

Programmed cell death protein 1 (PD-1) is found on activated T cells, while its ligand PD-L1 is mostly present on tumor cells. The PD-1/PD-L1 pathway helps cancer cells evade the immune response [17] and promotes tumor growth. PD-L1 levels are higher in TNBC than in non-TNBC and higher in CSCs [18], thereby aiding CSC expansion and immune evasion by suppressing T-cell activation. Anti-PD-L1 antibodies can inhibit TNBC growth and are FDA-approved for treatment [19]. However, TNBC shows resistance to immunotherapy, partly due to the abundance of CSCs and CAFs [20]. The mechanisms behind PD-L1 overexpression in CSCs and TNBC were previously unclear. Our research indicates that p38γ increases CXCL5, its receptor CXCX2, and PD-L1 in TNBC cells, creating a positive feedback loop that drives tumorigenesis (see Figure 1). Further studies show that knocking out p38γ in epithelial cells reduces CXCL5 and PD-L1 expression and fibrosis in PyMT tumors, as evidenced by immunohistochemistry and Sirius red staining for collagen II. This demonstrates that p38γ is essential for CXCL5 and PD-L1 expression, as well as for collagen metabolism in the stroma. RNA-seq analysis confirmed that epithelial p38γ knockout downregulates several pathways, including collagen metabolism, fibrosis (relevant to diseases like idiopathic pulmonary fibrosis treated with pirfenidone), CSC markers such as stahimim1, wound healing, and cytokine signaling. Notably, our findings suggest that epithelial p38γ knockout reduces collagen metabolism in breast cancers, possibly decreasing fibrosis and immune evasion. Thus, PD-L1 promotes CSCs and both p38γ and CXCL5 facilitate metastasis [7,8] while that p38γ directly increases PD-L1 and CXCL5 in normal mammalian epithelial cells (MCF10A).

Overexpression of p38γ raised PD-L1 and CXCL5 levels, though not CXCR2, indicating its necessity and sufficiency for their expression. Conversely, knocking down p38γ in TNBC cells reduced PD-L1 and CXCL5 levels. CXCL5 in the extracellular environment increased p-ERK l levels and enhanced p38γ-PD-L1 interactions in epithelial cells, with recombinant CXCL5 specifically boosting PD-L1 expression and interactions without affecting p38γ phosphorylation. CXCL5 induced PD-L1 upregulation in epithelial cells but [21] reduced it in CAFs, indicating a cell-specific regulatory mechanism. Kinetic studies showed that CXCL5 increased PD-L1 levels in epithelial cells at 3 and 6 h, but not in CAFs. p38γ promotes CSC expansion, which is linked to PD-L1-mediated oncogenesis in TNBC [20]. Since CXCL5 is upregulated during inflammation, its activation of PD-L1 via p38γ suggests that inflammatory signals can rapidly trigger this circuit, thereby promoting CSC growth and TNBC progression (see Figure 1). As PD-L1 encourages CSCs and TNBC advancement [21], and CXCL5 facilitates metastasis [8] and immune suppression [9], these findings imply that p38γ, together with PD-L1 and CXCL5, may collaboratively drive TNBC tumorigenesis by expanding CSCs and establishing an immunosuppressive tumor microenvironment.

## 2. Pancreatic Cancer

The role of p38γ in pancreatic ductal adenocarcinoma (PDAC): p38γ, unlike its isoforms, is specifically activated by mutant KRAS G12V but not by HRAS G12V transfection in GI epithelial cells. KRAS mutations (G12D and G12V) are present in up to 84% of PDAC cases. Mutant KRAS increases p38γ protein and RNA levels in intestinal epithelial cells, but not in murine NIH 3T3 fibroblasts. Our studies show elevated phosphorylated and total p38γ proteins and RNAs in KRAS-transformed HPNE (HPNE/KRAS) compared to vector-transfected cells [22]. This shows that oncogenic KRAS (G12D) activates p38γ in human pancreatic nestin-expressing cells (HPNE). Hence, p38γ is the only MAPK-activated by KRAS at both transcriptional and post-translational levels, indicating its role as a key effector of KRAS in PDAC [22].

Since aerobic glycolysis is a hallmark of cancer [23], p38γ can increase glucose uptake in fibroblasts by upregulating GLUT1. We found that KRAS transformation and p38γ overexpression boost the protein levels of the glycolytic activator PFKFB3 [24] and the glucose transporter GLUT2 (but not GLUT1). This shows that p38γ is necessary for KRAS-driven glycolytic pathways in PDAC cells. Silencing KRAS or p38γ suppresses this stimulation in KRAS-mutant PDAC cells. p38γ shRNA reduces growth in two KRAS-mutant human PDAC lines, and treatment with the p38γ inhibitor pirfenidone (PFD, 200 µg/mL) inhibits growth of KRAS-mutant PDAC cells but not normal HPNE cells. These findings demonstrate that p38γ is essential for activating the KRAS glycolytic pathway and for KRAS-driven proliferation in PDAC cells.

Oncogenic KRAS mutations occur in almost all cases of PDAC at the initiation stage, yet KRAS remains the most difficult target. Despite the initial benefit of KRAS-G12C inhibitors for patients with tumors harboring this mutation, the rapid emergence of drug resistance indicates that KRAS activity may be regulated by positive and negative feedback loops that are difficult to block with KRAS inhibitors. Moreover, there are still no pancreatic-dominant mutant KRAS (G12D or G12V) inhibitors approved for clinical trials. The KRAS oncogene signals through multiple effectors to meet the delicate demands of transformation, and it is therefore essential to identify a master effector of KRAS to activate this signaling network for intervention. While constitutive proliferative signaling of KRAS effectors is primarily responsible for PDAC oncogenesis, oncogene signaling must also crosstalk with the TME to program immune escape, thereby driving tumor growth and expansion. There is thus an urgent need to identify a master effector of the KRAS oncogene that both stimulates tumor cell growth and programs an immune-suppressive TME, as a target to block KRAS-driven PDAC tumorigenesis.

Normal cells rely on mitochondrial oxidative phosphorylation, whereas cancer cells switch to aerobic glycolysis (i.e., the Warburg effect) for energy production even in the presence of sufficient oxygen [25,26]. Accordingly, the KRAS oncogene activates glycolysis to generate ATP that supports constitutive proliferative signaling in cancer [23,27]. Therefore, cancer cells more efficiently take up extracellular glucose via glucose transporters (GLUTs) and secrete more lactate, resulting in an acidic TME [28]. This acidification is driven by several cellular rate-limiting glycolytic activators, including 6-phosphofructo-2-kinase-2/fructose-2,6-bisphosphatase-3 (PFKFB3). Glycolysis in cancer cells also activates host cells via secreted lactate, leading to an immunosuppressive TME [29]. Thus, metabolic reprogramming, a hallmark of cancer, not only endows cancer cells with proliferative potential but is also required for KRAS-induced tumor-stromal crosstalk via its metabolites.

The KRAS oncogene can also inhibit tumor immunity, creating an immunosuppressive TME that drives immune evasion. In tumor cells, KRAS activates cytokines and chemokines that mediate signaling crosstalk between tumor cells and host cells, such as CAFs, thereby inhibiting CD8^+^ T cell infiltration [30]. In addition, KRAS decreases major histocompatibility complex antigen presentation and upregulates PD-L1, thereby impairing antigen presentation and inhibiting T cell recognition, leading to immune evasion [31]. KRAS also upregulates specific members of the type I cytokine receptor family and stimulates expression of the cytokines IL-4 and IL-13 in PDAC cells [32]. These tumor-derived and secreted cytokines/chemokines may mediate KRAS oncogene signaling to program the immunosuppressive TME, regulate host cell responses, and promote PDAC tumorigenesis [27,32]. It is thus critical to investigate how tumor-derived cytokines/chemokines are regulated.

p38γ is a conserved inflammatory kinase that mediates KRAS oncogene signaling to activate metabolic programming and the inflammatory network, promoting PDAC tumorigenesis [33]. Our study shows that p38γ, in addition to phosphorylation, is activated by KRAS at both the protein and RNA levels in epithelial cells but not in fibroblasts, and is essential for RAS-driven transformation and PDAC development [22,34]. Moreover, p38γ enhances glucose uptake [35] and supports metabolic adaptation [36] while also upregulating several pro-inflammatory cytokines [37]. These findings suggest that epithelial p38γ transmits KRAS oncogenic signals in tumor cells, thereby generating ATP to promote proliferation and fostering an immunosuppressive tumor microenvironment through tumor-derived cytokines. This p38γ signaling pathway downstream of KRAS may promote tumor growth and help cancer evade immune responses, thereby driving PDAC.

We hypothesize that p38γ functions as a key KRAS effector that fuels tumor cell metabolism via glycolysis and facilitates immune evasion by boosting pro-inflammatory chemokine CXCL5 and PD-L1, transmitting KRAS signals from tumor to stromal cells. Targeting p38γ could inhibit KRAS-driven PDAC by disrupting these metabolic and inflammatory pathways. Our findings reveal that KRAS activates p38γ at both the phosphorylation and RNA/protein levels, and that p38γ is necessary and sufficient to activate the p-PFKFB3/PFKFB3/GLUT2 metabolic pathway in PDAC cells, which is vital for KRAS-dependent tumor growth in vitro. In rat intestinal epithelial cells, KRAS increases p38γ (but not other family members) at both protein and RNA levels [38], but in human pancreatic nestin-expressing cells (HPNE) increases phosphorylated [39] and total p38γ protein along with RNAs, indicating an important role of p38γ in promoting KRAS transformation.

To our knowledge, p38γ is the only MAPK activated by KRAS at both transcriptional and post-translational levels, indicating its role as a primary effector for KRAS in PDAC. Since cancer cells commonly undergo aerobic glycolysis [23], p38γ can stimulate glucose uptake by increasing GLUT1 expression in fibroblasts [40]. We explored whether p38γ is necessary for KRAS to activate glycolytic pathways in PDAC cells, and found that KRAS transformation and p38γ overexpression increase PFKFB3 (a glycolytic activator) and GLUT2 expression [22]. Silencing KRAS or p38γ suppresses this stimulation in KRAS-mutant PDAC cells. Furthermore, p38γ shRNA inhibits the growth of two KRAS-mutant human PDAC lines, and a pharmacologic p38γ inhibitor, pirfenidone (PFD, 200 μg/mL), reduces the growth of KRAS-mutant PDAC cells but not normal HPNE cells [22]. This demonstrates p38γ’s essential role in activating the glycolytic pathway and proliferation in PDAC. p38γ promotes PFKFB3/S467 phosphorylation, and pancreatic p38γ knockout inhibits tumor development and glycolysis by reducing p-PFKFB3/S467, cytokines, and α-smooth muscle actin (α-SMA), a stromal marker. Similarly, silencing PFKFB3 and GLUT2 depends on p38γ to inhibit glycolysis and tumor growth [22].

Since KRAS increases p-p38γ, we tested whether p38γ’s phosphorylation of PFKFB3 depends on KRAS. IP/WB experiments show p38γ binds PFKFB3 only in KRAS-transformed cells [41]. Mass spectrometry confirmed that p38γ phosphorylates PFKFB3 at S467, activating it, and that this phosphorylation is absent in S457A mutants [22]. Mice lacking p38γ in KPC models show significantly increased survival, decreased p-PFKFB3/PFKFB3/Glut2, cytokines IL-1α and IL-6, and α-SMA, indicating p38γ’s role in PDAC tumor growth, cytokine production, and stromal fibrosis via metabolic pathways. Additionally, p38γ knockout cells exhibit reduced extracellular acidification rate (ECAR) but not oxygen consumption rate (OCR), lower glucose uptake, PFK activity, and lactate secretion compared to control cells, confirming p38γ’s necessity for KRAS-induced glycolysis. Depleting PFKFB3 and GLUT2 inhibits glucose uptake and lactate production only in control KPC cells, not in p38γ knockout cells, further demonstrating their dependence on p38γ to promote glycolysis [22]. In summary, p38γ phosphorylates PFKFB3, which is crucial for KRAS-driven glycolytic activation, stromal fibrosis, cytokine release, and PDAC progression.

PFD depends on p38γ to inhibit KPC xenograft growth and decrease α-SMA expression, and its inhibitory activity against pancreatic tumorigenesis in KTC mice is coupled with a reversal of immune evasion. Epithelial p38γ KO also inhibits PDAC tumorigenesis in KTC mice, again reinforcing the essential role of p38γ in KRAS tumorigenesis regardless of tumor suppressors. Next, we assessed the effects of its pharmacological inhibitor, PFD, on KPC xenograft growth in nude mice. Notably, the same treatment significantly inhibits KPC but not KPC/p38γ KO tumor growth, a pattern that correlates with suppression of the fibroblast marker α-SMA. Although α-SMA is a well-established stromal myofibroblast marker with prognostic value in PDAC, deletion of α-SMA^+^ myofibroblasts in B6 mice leads to increased KPC tumorigenesis by an unknown mechanism.

PFD is a clinically approved anti-fibrosis agent and its therapeutic effects are observed only in KPC, not in KPC/p38γ KO xenografts, in association with p38γ-dependent suppression of α-SMA expression, indicating that epithelial p38γ is required for tumor growth and stromal fibrosis. PFD therapy also significantly increases the survival of primary KTC mice. To assess the regulatory effects of PFD on immune landscapes, tumor-associated immune cells were quantified by flow cytometry. Results showed that PFD treatment significantly increased tumor-infiltrating CD3^+^ and CD4^+^ T cells and moderately reduced neutrophil (Ly6G^+^) infiltration. Since CD4^+^ T cells can eradicate tumor cells, these results are consistent with the KTC growth-inhibitory activity of PFD, likely by increasing CD3+ and CD4+ T cell infiltration, indicating a reversal of immune evasion.

PDAC is characterized by a dense desmoplastic stroma that constitutes up to 90% of the tumor mass and plays crucial roles in disease progression, immune evasion, and therapy resistance. Oncogenic KRAS-activated p38γ, implicated in extracellular matrix (ECM) remodeling, can be pharmacologically inhibited by the FDA-approved antifibrotic drug pirfenidone (PFD). Thus, targeting p38γ, a mediator of ECM remodeling, could reduce fibrosis-associated chemotherapy resistance and immune exclusion in PDAC patients. We aim to further examine the effects of epithelial p38γ KO on ECM signaling in the KTC tumor, which is associated with activated TGFβ signaling. Results showed that epithelial p38γ KO significantly decreased collagen deposition in the ECM of KTC tumors, and similar effects were observed after PFD therapy. Although significant inhibition of α-SMA expression was not achieved, both p38γ KO and PFD therapy markedly reduced staining. This strongly indicates that epithelial p38γ is required for KRAS-mediated stromal activation by modulating ECM signaling. Thus, p38γ may signal in the tumor and stroma by promoting cancer cell growth, stimulating fibrosis, programming immune evasion, and thereby promoting PDAC tumorigenesis.

PFD and PFK15 cooperatively decreased p-PFKFB3 protein levels and inhibited glucose uptake, lactate secretion, and colony formation in KPC, but not in KPC/p38γ KO cells. This clearly shows that PFD and the PFKFB3 inhibitor PFK15 depend on p38γ to cooperatively block the metabolic pathway and KPC growth [22]. Importantly, the combination in mice significantly suppressed the growth of KPC but not KPC/p38γ KO xenografts, whereas either alone failed to do so. This clearly indicates that p38γ is the sole target of the PFD/PFK15 combination for inhibiting glycolysis and KPC growth in cells and mice, even though PFKFB3 is also expressed in host cells, such as endothelial cells [42]. Because p38γ is required for KRAS-activated PFKFB3 and CXCL5 expression in PDAC cells and tumors, the moderate antitumor effect of the PFD/PFK15 combination in KPC but not in KPC/p38γ KO tumors indicates that a more complete blockage of KRAS-dependent tumor growth may be achieved by adding other inhibitors of p38γ-activated pathways, such as CXCL5 and PD-L1.

**Figure 2:**
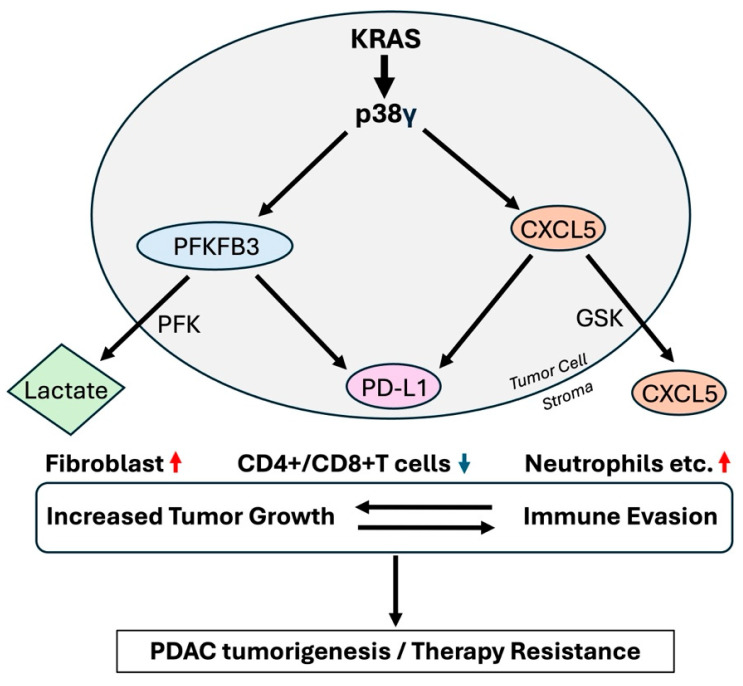
The p38γ/CXCL5/PD-L1 signaling loop in PDAC tumorigenesis. Targeting the p38γ network will be an effective strategy to block PDAC tumorigenesis and therapeutic resistance. The models show that epithelial p38γ cooperates with PFKFB3, PD-L1, and CXCL5 to activate fibrosis, inhibit infiltration of CD4^+^ and CD8^+^ T cells, and increase neutrophil recruitment, thereby promoting tumor growth and immune evasion and driving PDAC tumorigenesis.

Gemcitabine (GEM) is an important chemotherapeutic agent for PDAC, and stromal activation is a major mechanism [43] of resistance. Studies have shown that co-injecting cancer cells with fibroblasts or HPSC can enhance the growth-inhibitory activity of PFD when combined with GEM [44]. Here, PFD increases GEM-induced growth inhibition in two human PDAC cell lines. Moreover, co-culturing primary human PDAC organoids with primary fibroblasts (isolated from the same patient) increases sensitivity to PFD, with the IC_50_ decreasing by almost two-fold. These results indicate that PFD holds great promise in PDAC therapy by targeting tumor-promoting p38γ signaling in both cancer cells and CAFs. Because p38γ is required for activation of the PFKFB3/CXCL5/CXCR2/PD-L1 pathway, combined targeting of these pathways should be more effective in inhibiting KRAS-dependent PDAC growth. To determine whether p38γ is a therapeutic target in human PDAC, a cohort of pathological specimens was analyzed for p38γ expression by IHC. p38γ is overexpressed in PDAC samples compared with normal tissues, and higher p38γ levels predict decreased patient survival. Together, our preliminary studies demonstrate that p38γ is a master effector of KRAS, driving PDAC tumorigenesis, fibrosis, and therapy resistance by stimulating PFKFB3-driven glycolysis, CXCL5-dependent inflammation, and PD-L1-induced immune evasion (Figure 2).

## 3. Colorectal Cancer (CRC)

Colorectal cancer (CRC) is the second leading cause of cancer death in the USA. Inflammation is causally linked to CRC [45]. For example, more than 20% of patients with inflammatory bowel disease (IBD) will develop colitis-associated cancer (CAC), with a mortality rate of 50% [46]. In addition to extrinsic conditions, intrinsic genetic events, such as mutations in the tumor suppressor gene adenomatous polyposis coli (APC), which occur in up to 80% of sporadic CRC cases, elicit an atypical inflammatory response [47,48,49]. The KRAS oncogene mutation is detected in up to 40% of CRC cases and is associated with an inflammatory tumor microenvironment [50]. Although nuclear factor-κB (NF-κB) and signal transducer and activator of transcription 3 (Stat3) play established roles in promoting inflammation-induced CR [45], both are transcription factors rather than drug targets. There is a critical need to identify druggable inflammatory targets that link common CRC risk factors to CRC tumorigenesis, enabling therapeutic intervention [45,48,49].

The Wnt/β-catenin pathway is aberrantly activated in 90% of CRC [51,52]. β-catenin, a crucial transcriptional co-activator of Wnt signaling through interaction with T-cell factor (TCF), is stabilized and activated by Wnt ligands. In the absence of the ligands, β-catenin is phosphorylated by glycogen synthase kinase-3β (GSK3β) at its N-terminus, resulting in its degradation through the coordinated action of casein kinases (CK), APC, and Axin (together called the “destruction complex”) [53,54]. When APC is mutated, β-catenin is released from the complex and translocated to the nucleus, leading to unchecked Wnt transcription and CRC tumorigenesis [54,55]. In contrast to the N-terminus, β-catenin is also phosphorylated at its C-terminus, which leads to its stabilization; however, the responsible kinases are largely unknown [54,56]. Recent studies suggest an important role for Wnt/β-catenin signaling in programming immunosuppression in the tumor microenvironment (TME). Identification of a novel β-catenin kinase that can be activated by both inflammatory and genetic factors may be essential for targeting Wnt signaling to block its proliferative signaling and to activate its anti-tumor immunity [53,57].

p38 mitogen-activated protein kinases (MAPKs, including the p38α, β, γ, and δ isoforms) are a family of highly conserved proteins that can be activated by inflammatory and stress stimuli to regulate the expression of cytokines, inflammatory mediators, and survival genes through an isoform-specific mechanism [55,58]. In contrast to its family member p38α, p38γ promotes transformation and invasive/metastatic responses. p38γ is activated by KRAS in epithelial cells and is required for Ras transformation by activating its effector pathways [2,59,60]. Moreover, p38γ increases pro-inflammatory cytokine expression [61] and is required for inflammation-induced colon cancer [61]. Furthermore, p38γ is upregulated in several human malignancies of epithelial origin [60,62]. Together, these results indicate that intestinal epithelial p38γ may link extrinsic and intrinsic oncogenic signaling to proliferative Wnt pathways and pro-inflammatory cytokines/chemokines, thereby promoting intestinal tumorigenesis by driving immune evasion.

The study of p38γ kinase as a major link between common CRC risk factors and immune evasion is highly innovative, as it suggests that targeting a kinase could be an effective approach to colon cancer therapy by disrupting the tumor–TME interaction. The therapeutic potential is further supported by recently reported promising results from a phase II clinical trial using the p38γ inhibitor pirfenidone [34,63] in combination with an anti-PD-L1 antibody in patients with colon cancer. Thus, targeting p38γ pathways may be a highly effective therapy for CRC by awakening anti-tumor immunity.

**Figure 3:**
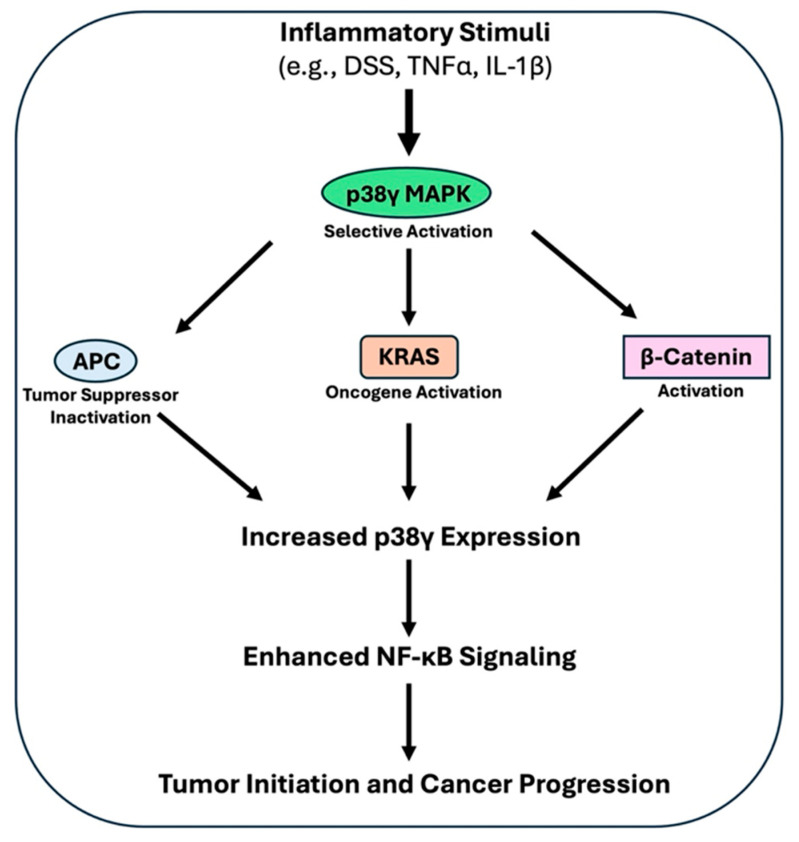
The p38γ/KRAS/β-catenin signaling loop in CRC tumorigenesis. p38γMAPK is selectively activated by inflammation or by inflammatory stimuli such as dextran sulfate sodium (DSS), tumor necrosis factor-alpha (TNFα), or interleukin-1 beta (IL-1β). This, in turn, drives KRAS oncogene activation (but not HRAS) and inactivates the APC tumor suppressor. KRAS, but not HRAS, selectively induces p38γ expression in intestinal epithelial cells. p38γ is elevated in colon tissues of mice, in parallel with activated β-catenin. Upregulated p38γ in the intestine enhances NF-κB signaling, which in turn initiates inflammation in inflammatory colon tumors and promotes cancer progression.

We tested if p38γ is selectively activated in cells and/or tissues by KRAS oncogene activation, APC tumor suppressor inactivation, and inflammation. CRC is frequently associated with inflammatory bowel disease (IBD), whereas common genetic pathways, such as those involving Wnt, β-catenin, KRAS mutations, and adenomatous polyposis coli (APC) inactivation, can differ between CRC and colitis-associated cancer (CAC) [46]. The mutant KRAS (G12V) increases endogenous p38γ, but not p38α and p38δ, protein expression in IEC-6 cells but not NIH 3T3 cells, whereas the mutant HRAS increases p38γ in both IEC-6 and NIH 3T3 cells, indicating that in intestinal IEC-6 cells, the KRAS oncogene selectively activates the p38γ stress kinase. Inflammation stress stimuli (DSS and TNFα) also activate p38γ, but not p38α or p38δ in colon cancer cells and in colitis/tumor tissues of IL-10^-/-^ and APC^min/+^ tissues as compared to their sex/age-matched normal controls. Importantly, p38γ staining is increased in colon tissues of UCD and CAC of clinic CRC specimens as compared to normal control. These results together indicate that p38γ is selectively activated by inflammation, KRAS activation, and inactivation of the tumor suppressor APC in intestinal cells and/or tissues, where it may transduce its oncogenic signaling to promote tumorigenesis (Figure 3).

β-catenin can be phosphorylated at several MAPK phosphorylation motifs, S191, S248, and S605, to regulate its localization in bone marrow-derived cells. We mutated these three residues to Alanine (A) and analyzed their in vitro phosphorylation by incubating Flag precipitates with His-p38γ and a specific p-S/TP antibody (recognizing a protein motif phosphorylated by a MAPK). β-catenin/S605A almost completely lost the reactivity as compared with WT and other mutants, indicating that p38γ specifically phosphorylates β-catenin/S605 in vitro. Additional experiments showed that p38γ phosphorylates β-catenin/S605 as detected with a phospho-specific antibody, and β-catenin, not its S695A mutant, is stabilized by co-transfected constitutive active (CA) p38γ. Furthermore, CA p38γ overexpression alone in HCT116 cells increases p-β-catenin/S605 (p-S605) and Wnt targets but decreases p-S605 in S605A knock-in cells in which TCF4 also fails to interact with p38γ, indicating a critical role of the S605-coupled p38γ/β-catenin/TCF4 in Wnt transcription.

Both p38γ conditional knockout (KO) and treatment with the p38γ inhibitor pirfenidone (PFD) decreased the tumor score, indicating an essential role of p38γ in CAC. Cytokines and chemokines are important molecules that regulate immune cell infiltrations, and we next performed antibody arrays to screen for specific cytokines/chemokines that can mediate p38γ oncogenic signaling. Among several downregulated cytokines/chemokines, CXCL13 is the most substantially decreased chemokine in tumors of IL-10-/-/p38γ KO mice and of IL-10-/- mice treated with PFD. Moreover, similar decreases in CXCR5 and CXCL13 were demonstrated in HCT-116 human colon cancer cells by p38γ silencing in mouse colon cancer tissues. Consistent with the effects on CXCL13 and CXCR5 in colon tumors of IL-10-/- mice, WB analysis further showed decreased protein levels of β-catenin, p-β-catenin (p-β-catenin/S605), and several Wnt target genes following p38γ KO or treatment with the p38γ inhibitor PFD. These results together indicate that p38γ activates the Wnt/β-catenin pathway and CXCL13/CXCR5 axis and promotes CAC in IL-10-/- mice. p38γ KO and treatment with PFD reduce tumor score in AOM/DSS mice, decrease β-catenin and p-S605 levels, Wnt target gene expression, and cancer-like stem cell (CSC) markers. In addition, p38γ overexpression elevates β-catenin and CSC markers, whereas its silencing in human colon cancer cells decreases β-catenin levels and inhibits xenograft growth. These results together demonstrate that intestinal p38γ is required for Wnt/β-catenin and CSC signaling, as well as for CRC tumorigenesis and growth.

p38γ is required for CAC development in mice and for human colon cancer growth in nude mice by maintaining Wnt/β-catenin signaling. Studies in the AOM/DSS model were previously published [61] with colon tissues analyzed, tumor quantitation, RNA-seq, and WB. Results in the bottom panel were from IEC-6 cells, showing that p38γ overexpression activates Wnt/β-catenin, whereas those in the top panel showed that p38γ silencing inhibits β-catenin expression and xenograft growth in nude mice [61]. p38γ promotes polyp formation, activates Wnt/β-catenin signaling, and inhibits CD8^+^ T cell and macrophage infiltration in APC^min/+^ mice. APC^min/+^ is a well-established mouse model of colon cancer for studying Wnt/β-catenin signaling [64] and is also used to assess the effects of p38γ KO and inhibition on tumorigenesis. Tumor-associated macrophages (TAMs) can foster immunosuppression by releasing chemokines and cytokines that compromise the local immune response and may also suppress T cell functions by regulating other immune cells. The increase in both CD8^+^ T cell and macrophage infiltration in epithelial p38γ KO, but not in response to treatment with the p38γ inhibitor PFD, may result from p38γ-regulated chemokines and a potential off-target effect of PFD in the APC^min/+^ tumor model. p38γ activates Wnt/β-catenin signaling, increases Wnt-dependent PD-L1 expression [65], and inhibits CD8^+^ T cell/macrophage infiltration, suggesting that p38γ may promote tumorigenesis in APC^min/+^ mice by driving immune evasion.

PFD enhances the tumor-suppressing effects of anti-PD-L1 in the AOM/DSS model by boosting CD8^+^ T cell and macrophage infiltration in C57B/C6 mice. p38γ activity is essential for PD-L1 expression in mouse colon cancer, suggesting that inherent p38γ activity may contribute to immune resistance. The p38γ inhibitor PFD can counteract this resistance; the growth-inhibitory effect of the checkpoint inhibitor (CPI) in the AOM/DSS model, with and without PFD, affects tumor formation and protein levels. Anti-PD-L1 alone did not significantly increase CD8^+^ T cell or macrophage infiltration nor reduce tumor growth, indicating intrinsic resistance to immunotherapy. Notably, PFD boosted the anti-PD-L1’s effectiveness, reduced Wnt targets, PD-L1, β-catenin, and p-β-catenin levels, and increased immune cell infiltration. These findings suggest that intrinsic p38γ may confer resistance to CPI, a resistance that can be overcome by PFD, which promotes infiltration by CD8^+^ T cells and macrophages.

The role of p-β-catenin/S605 (S605) in p38γ activation of Wnt signaling and colon cancer growth was studied by stably expressing the mutant β-catenin/S605A (m605A) or an empty vector in HCT116 human colon cells by knock-in. Cells were pulsed with DSS and assessed for activation of the p38γ/β-catenin/Wnt target/CXCL13/CXCR5 pathway. DSS treatment increases levels of each molecule in this pathway in Vector (control) cells but not in m605A (mutant) cells, indicating an essential role of β-catenin/S605 in DSS-induced CXCL13/Wnt signaling. Moreover, p38γ overexpression in vector cells elevates p-β-catenin/S605 and c-Myc levels and promotes their binding to β-catenin and TCF4. p38γ overexpression also increases levels of total and phosphorylated β-catenin at S605 and increases colony formation in Vector but not in mutant cells, which correlates with increased Wnt target gene expression (c-Myc, cyclin D1, and CD44), indicating that S605 is required for p38γ to activate Wnt transcription and increase colon cancer growth.

**Figure 4:**
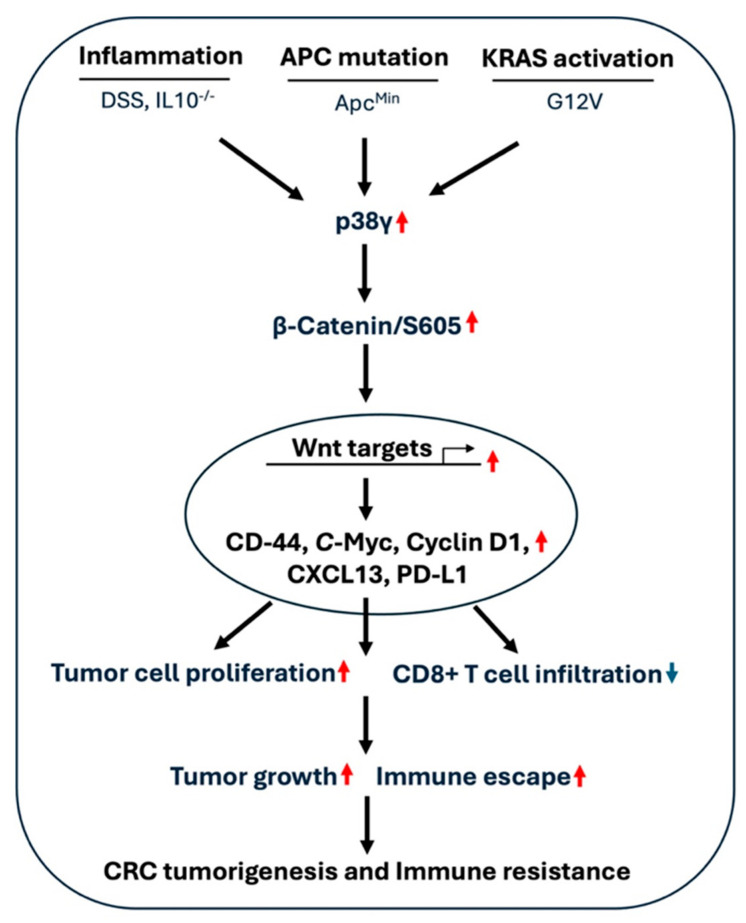
The p38γ/CXCL13/PD-L1 signaling mechanism in CRC tumorigenesis. p38γ is activated by inflammation and functions as an oncogene in colon cancer. Activated p38γ phosphorylates β-catenin at S605 and transcribes Wnt target genes, including CXCL13 and PD-L1, resulting in increased tumor cell proliferation and reduced CD8+ T cell infiltration, leading to immune escape and increased CRC tumorigenesis.

The role of endogenous p38γ in 5-FU-induced growth inhibition was further demonstrated in colon cancer cells for its links to Wnt/β-catenin signaling and chemotherapy resistance. At a higher 5-FU concentration (10 μM), PFD significantly enhanced 5-FU’s growth-inhibitory effect; this effect was reversed when p38γ was depleted, suggesting that PFD boosts 5-FU’s growth inhibition by targeting endogenous p38γ. Supporting this, both p38γ silencing and PFD treatment reduced cyclin D1 and TCF4 protein levels, which were also decreased by 5-FU. Additionally, PFD specifically downregulated p-S605, an effect reversed by p38γ depletion. These findings indicate that p38γ siRNA, PFD, and 5-FU lower Wnt target and TCF4 expression, with PFD likely inhibiting β-catenin/S605 phosphorylation mainly through p38γ suppression. Immunohistochemical assays of clinical specimens revealed elevated p38γ protein levels in human CRC tissues compared with normal controls. The TCGA database also showed higher *MAPK12* (p38γ) expression in colon cancer tissues than in normal tissues, and this was correlated with poorer overall and disease-free survival. Therefore, p38γ is activated in clinical colon cancer and serves as a poor prognostic marker. It promotes tumorigenesis by transmitting extrinsic and intrinsic oncogenic signals to activate the Wnt/β-catenin pathway and facilitates immune evasion by reducing infiltration by CD8^+^ T cells and macrophages (see Figure 4). Targeting p38γ signaling could offer a novel therapeutic strategy to restore antitumor immunity.

## 4. Future Perspectives

The future direction of this field should focus on establishing p38γ as both a mechanistic biomarker and a clinically actionable therapeutic target across inflammation-driven cancers. Although substantial evidence now supports the role of p38γ in regulating oncogenic signaling, metabolic reprogramming, fibrosis, cancer stemness, and immune evasion, additional studies are needed to define its isoform-specific substrates, context-dependent signaling networks, and interactions within the tumor microenvironment. Particular emphasis should be placed on understanding how p38γ coordinates crosstalk between tumor cells, stromal fibroblasts, and immune populations to promote therapy resistance and metastatic progression. The development of highly selective p38γ inhibitors, together with predictive biomarkers for patient stratification, will be critical for successful clinical translation. Moreover, rational combination strategies that incorporate p38γ inhibition with immune checkpoint blockade, chemotherapy, metabolic inhibitors, anti-fibrotic agents, and Wnt/β-catenin-targeted therapies may yield synergistic anti-tumor effects, particularly in refractory malignancies such as TNBC, PDAC, and CRC. Emerging technologies, including single-cell sequencing, spatial transcriptomics, patient-derived organoids, and genetically engineered mouse models, will further clarify the dynamic role of p38γ in tumor evolution and immune remodeling. Collectively, continued investigation of p38γ signaling has the potential to establish a new therapeutic paradigm that simultaneously targets tumor proliferation, stromal activation, metabolic adaptation, and immune suppression in inflammation-associated cancers.

## 5. Conclusions

In summary, this review identifies p38γ (MAPK12) as a central oncogenic and inflammatory kinase that integrates metabolic reprogramming, Wnt/β-catenin activation, fibrosis, cancer stemness, and immune evasion across triple-negative breast cancer (TNBC), pancreatic ductal adenocarcinoma (PDAC), and colorectal cancer (CRC). These three malignancies were selected because they represent distinct yet overlapping cancer paradigms in which p38γ-regulated pathways play critical roles: TNBC as a cancer driven by stemness and immune suppression; PDAC as a KRAS-dependent, highly desmoplastic malignancy; and CRC as an inflammation- and Wnt-driven disease. Importantly, the oncogenic functions of p38γ are not limited to these tumor types. Increased p38γ expression has also been reported in bladder cancer, where it correlates with advanced disease stage and poor patient prognosis, in nasopharyngeal carcinoma, where it promotes tumor growth and suppresses apoptosis, and in other malignancies, including hepatocellular carcinoma and gliomas. Collectively, these findings support a broader role for p38γ as a conserved regulator of cancer progression across diverse tissue types. The evidence reviewed here highlights p38γ as a master signaling hub linking inflammatory and oncogenic pathways and underscores its potential as a therapeutic target that can simultaneously suppress tumor growth, stromal activation, metabolic adaptation, and immune evasion across multiple cancers.

## Data Availability

The data presented in this study are available upon request from the corresponding author upon reasonable request.
